# An Industrial Scale Synthesis of Adipicdihydrazide (ADH)/Polyacrylate Hybrid with Excellent Formaldehyde Degradation Performance

**DOI:** 10.3390/polym11010086

**Published:** 2019-01-08

**Authors:** Rui Zhu, Renjie Chen, Yunxia Duo, Saigang Zhang, Delong Xie, Yi Mei

**Affiliations:** 1 Faculty of Chemical Engineering, Kunming University of Science and Technology, Kunming 650500, China; zhurui412@outlook.com (R.Z.); crj1011@126.com (R.C.); duoyunxia949@163.com (Y.D.); zhangsaigang@126.com (S.Z.); 2 The Higher Educational Key Laboratory for Phosphorus Chemical Engineering of Yunnan Province, Kunming University of Science and Technology, Kunming 650500, China

**Keywords:** adipicdihydrazide, formaldehyde degradation, polyacrylate latex, hybrid film

## Abstract

A simple and versatile route for industrial scale synthesis of adipicdihydrazide (ADH)/polymer hybrids with excellent performance of formaldehyde degradation is proposed in this paper. The ADH compound is uniformly dispersed in poly(methyl methacrylate-butyl acrylate-methacrylic acid) (P(MMA-BA-MAA)) latex, which is validated by UV and dispersibility tests. The results illustrate that ADH has excellent compatibility and dispersion stability without affecting the film formation of the polymer latex. Furthermore, scanning electron microscope (SEM) and mapping analysis of the hybrid films also demonstrate that ADH is homogenously dispersed in the polymer matrix. Compared with neat polymers, the thermal properties of hybrid films are improved, for example, *T*_0.5_ increases by 8.3 °C. According to qualitative tests of the 4-amino-3-hydrazino-5-mercapto-1,2,4-triazol-red/green/blue (AHMT-RGB) method, the hybrid films demonstrate high formaldehyde removal efficiency. On the basis of the semi-quantitative test of Fourier Transform infrared spectroscopy (FTIR) measurements, the rate of formaldehyde degradation can reach 1.034 × 10^2^ mol/(h·m^3^) for the hybrid film with 5 wt% ADH.

## 1. Introduction

Formaldehyde, characterized by its chemical reactivity and quality, as well as competitive price, is an important precursor material that is widely used in organic synthesis processes. However, free formaldehyde is also a serious air pollutant, which may cause respiratory irritation in humans at concentrations greater than 1.0 ppm. [1] Free formaldehyde is mainly produced from the following three sources: solid wood (furniture), medicine, and food [1,2,3,4,5]. With increased improvement in living conditions, a premium is imposed on the importance of a healthy environment, including the level of indoor free formaldehyde [6,7,8,9,10]. Therefore, degradation of free formaldehyde in habitable spaces is of great significance.

There are various technologies for degrading unbound formaldehyde, including physical adsorption [11], catalytic oxidation [12], and chemical reactions [13]. Physical adsorption is the most common method to remove aldehydes in industry, where formaldehyde is fixed on the adsorbent surface by van der Waals force. Renggaa et al. [14] investigated the performance of silver nanoparticles adsorbed in bamboo based activated carbon (Ag-AC) using a continuous fixed-bed reactor for formaldehyde removal. The results showed that the rate of formaldehyde removal by the Ag-AC reactor was 2.36 times higher than that by the AC reactor. Although the adsorption effect of activated carbon has been improved, there is still a problem of desorption. More importantly, the high price of nano silver has greatly increased the cost of composite materials, which is a challenge to industrialization. In the catalytic oxidation method, formaldehyde and oxygen are captured by the hydroxyl group on the surface of metal oxide [15]. Different catalysts have been introduced to enhance the reaction of formaldehyde degradation, where many inorganic materials with high reactive index are utilized for catalytic oxidative degradation of formaldehyde [16]. Wang and coworkers [17] prepared an active Pt-TiO_2_ composite catalytic material by deposition precipitation methods with reduction processes. The degradation rate of formaldehyde (HCHO) was measured by a photoacoustic field Gas-Monitor-INNOVA 1412. Compared with the pure TiO_2_, higher photocatalytic activity of the Pt-doped TiO_2_ was observed under visible light. Hu et al. [18] successfully synthesized TiO_2_/Hydroxyapatite (HAP) nanocomposites by a simple hydrothermal method. They revealed an affinity between HAP and formaldehyde via the weak chemical bond, which was testified by van’t Hoff plot based on the Langmuir–Hinshelwood model. The experiment was conducted in a fixed-bed annular quartz reactor with a heater around and an air-cooling system, and the degradation rate of HCHO was tested by Agilent-4890 GC system (Agilent, Santa Clara, CA, USA). Catalytic oxidation solves the problem of desorption compared with physical adsorption degradation. However, some inorganic materials, such as HAP, have a large refractive index, and some precious metal-loaded catalytic materials, such as Pt/TiO_2_, are darker in color, which limits their application in varnishes and interior wall coatings with whiteness requirements. In comparison with other degradation technologies, the chemical reaction degradation technology has individual superiority. Formaldehyde could be adsorbed and immobilized via the nucleophilicity interaction with the oxygen atom on the unsaturated carbonyl group [19], such as Mannich reaction, Cannizzaro reaction, Reppe reaction, Prins Cyclization, Aldol condensation reaction, and other chemical reactions [20]. The oxygen atom on the aldehyde group has a partial negative charge, which is strongly nucleophilic and easily reacts with the electrophilic reagent. Thus, a nucleophilic addition reaction is likely to occur in the carbonyl [21,22,23]. The amino group is highly reactive and oxidative [24], which improves the formaldehyde degradation ability of the polymer, while keeping its mechanical properties and other properties [25,26,27]. Therefore, formaldehyde desorption is suppressed and its engineering application range is extended. Amino groups containing the amino modified compounds are of key importance to maintaining the desired performance of the polymer when added to a matrix. This has drawn extensive attention in the field of degradable polymer latex.

It is well known that formaldehyde degradation of glutathione comprised of an amide bond can be achieved by chemical reaction. Xu et al. [28] compared the results of diverse exploratory experiments, and pointed out glutathione existing on the surface of plants, which could be coupled with formaldehyde to first form S-hydroxymethyl glutathione, and then be converted into S-formyl glutathione [29,30]. The degradation process was pollution-free based on the fact that formaldehyde dehydrogenase plays a vital role. However, the degradation efficiency declined quickly owing to the short activity period of dehydrogenase. Moreover, formaldehyde reacted with the active methylene group in the weak base, producing α-β unsaturation by Knoevenagel condensation reaction. Guo et al. [31] synthesized 3,5,5,7-tetraacetyl-2,8-nonanedione by integrating 2,4-pentanedione and formaldehyde at room temperature for three days, and studied the reaction mechanism. Although the efficiency of the degradation was strengthened, the obtained product was yellow, which limited its applications in the polymer latex. Moreover, formaldehyde contains alpha hydrogen group, leading to the Mannich reaction. Photong et al. [32] used 3-aminopropyltrimethoxysilane (APTMS) to modify TiO_2_ by the combined technique of peroxotitanic acid (PTA) and sol-gel dip-coating method. Although its efficiency was up to 92% after seven cycles, the PTA reflow time had a great impact on the degradation efficiency. Ma et al. [33] modified activated carbon (AC) with hexamethylenediamine (HMDA) by chemical method. The introduction of HMDA improved the formaldehyde degradation performance of AC by providing chemical adsorption sites. Nevertheless, the modification decreased the number of pores on the AC surface, which suppressed the degradation of formaldehyde. In addition to the above, the formaldehyde could also react with hydrogen on the benzene ring of phenolic compounds by nucleophilic addition reaction. To understand the formaldehyde degradation characteristics of tea polyphenol, Yu et al. [34] studied various related factors. The results indicated that the formaldehyde was removed more completely by tea polyphenol with higher pH, higher reaction temperature, and longer reaction time. Also, it was found that the tea polyphenol was not a great formaldehyde scavenger owing to its high cost of preparation.

As mentioned above, the functional materials modified by amine group have been proven to be effective for formaldehyde degradation. However, reports of simple, practicable synthetic methods of amino/polymer hybrid film, for example, physical blending, are rather scarce. It is the first time that a mixture of adipicdihydrazide (ADH) and poly(methyl methacrylate-butyl acrylate-methacrylic acid) (P(MMA-BA-MAA)) latexes with an outstanding homogeneous dispersion property has been reported by cell pulverization. Moreover, it can be further used to produce functional water-borne coatings for formaldehyde degradation. The ADH has great compatibility with water-borne polymers, as well as eminent aldehyde removal performance. The obtained latex will be used as the binder of functional waterborne wood coatings to inhibit the source of formaldehyde release, as well as interior wall coatings where a large amount of pores exist as a result of the excessive fillers added during the coating preparation process. Free formaldehyde in the air can fully contact with the amine groups in the coatings throughout the pore structure, thereby removing most of the free formaldehyde indoors. The dispersion stability of ADH/P(MMA-BA-MAA) hybrid latex is characterized with UV–Vis transmission spectra. The SEM results reveal that the surface of polymer films with and without ADH is smooth. ADH is dispersed evenly according to the mapping analysis. The thermal properties of the hybrid films are conducted by Thermo Gravimetric Analysis (TGA) and Differential Scanning Calorimeter (DSC), and it is shown that the thermal properties have been improved, for example, *T*_0.5_ increases by 8.3 °C. The formaldehyde degradation efficiency of hybrid film is qualitatively measured using the AHMT method and semi-quantitatively measured by FTIR.

## 2. Materials and Methods

### 2.1. Raw Material

Adipicdihydrazide (99%) used in the paper was from Shanghai Aladdin Biochemical Technology Co., Ltd. (Shanghai, China). Commercial methyl methacrylate (MMA, 99%), butyl acrylate (BA, 99.8%), and methacrylic acid (MAA, 99.5%) were provided by Beijing J&K Scientific Ltd. (Beijing, China), and the inhibitors were removed before usage. Sodium dodecyl sulfate (SDS, 92.5%–100.5%) was offered by Tianjin Feng chuan Chemical Reagent Science and Technology Co., Ltd. (Tianjin, China). Hexadecane (HD) and potassium persulfate (KPS, 99.5%) were provided by Shanghai Mclean biochemical science and Technology Co., Ltd. (Shanghai, China). Deionized water was obtained from the laboratory of researchers.

### 2.2. Synthesis of Acrylic Emulsion

Emulsion polymerization of acrylic ester (MMA + BA) and methacrylic acid (MAA) was carried out by semi-continuous emulsion polymerization. Firstly, emulsifier was added into DI water in a 250 mL four-neck round-bottomed flask in water bath, which was equipped with reflux condenser and stirrer, to form aqueous phase (i). Meanwhile, co-stabilizer was dissolved in monomer to form the oil phase and initiator was dissolved in deionized (DI) water to form the aqueous phase (ii). The oil phase and the aqueous phase (ii) were then slowly added into the aqueous phase (i) when the temperature was heated to 80 °C. Seed emulsion was gradually formed in fifteen minutes after the addition of monomer and initiator. After fifteen minutes, the oil phase and the aqueous phase (ii) were continuously added simultaneously into the aqueous phase (i). The mixture was heated up from 80 to 85 °C after the completion of the oil phase and the aqueous phase (ii), and was kept it at 85 °C for one hour. The recipe adopted for the synthesis of the polymer latex is listed in Table 1.

### 2.3. Preparation of ADH/P(MMA-BA-MAA) Hybrid Latex

Five grams of ADH compounds was dissolved in 50 mL of deionized water using ultrasonication. ADH solution of 0, 1, 2, and 3 g, respectively, was weighed and added into the given polymer latex (10 g) to obtain hybrid latex composed of 0%, 1.0%, 2.0%, and 3.0% (wt) of ADH, respectively, considering a polymer content of latex being 40%, and the ADH loading was 0%, 2.5%, 5%, and 7.5% (wt) of polymer, respectively. The hybrid latex was stirred for 5 min with Magneto and smashed for 30 min with a cell pulverizer (output power: 600 W, running period: 1.5 s, pause period: 5 s).

### 2.4. Preparation of ADH/P(MMA-BA-MAA) Hybrid Films

**Scheme 1:**
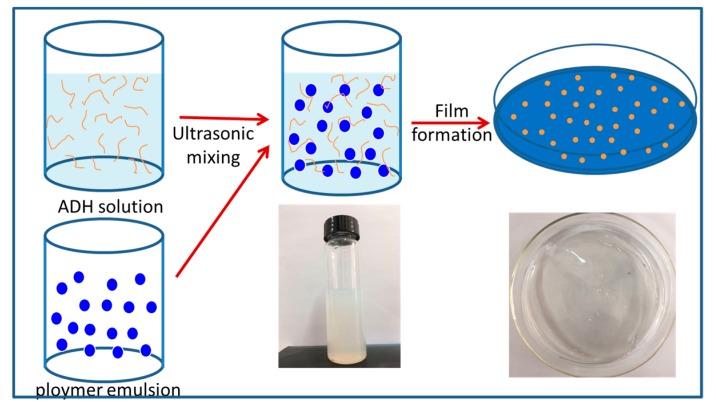
A schematic diagram of preparation of adipicdihydrazide (ADH)/poly(methyl methacrylate-butyl acrylate-methacrylic acid) (P(MMA-BA-MAA)) hybrid films.

The dispersed ADH/P(MMA-BA-MAA) hybrid emulsions were placed on petri dishes marked by number. They were then dried in a vacuum oven at 50 °C for 48 h to remove water. The preparation process of ADH/P(MMA-BA-MAA) hybrid films is shown in Scheme 1.

### 2.5. Characterization and Instruments

#### 2.5.1. Experimental Procedure of Formaldehyde Degradation

**Figure 1:**
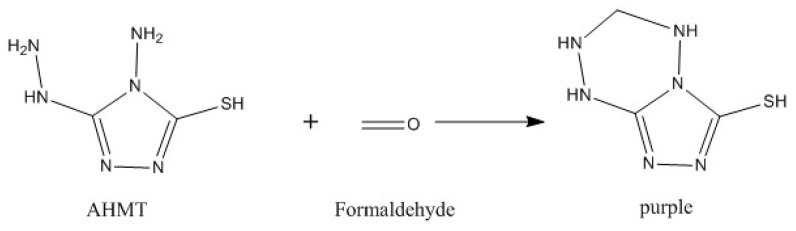
The principle of the AHMT method for detecting formaldehyde.

The AHMT reagent is white crystal at room temperature. When reacted with free formaldehyde in alkaline condition, its product is purple. With excessive AHMT, the color is darkened with an increasing content of free formaldehyde. On the basis of this principle (as shown in Figure 1), AHMT could be used to qualitatively measure the residual formaldehyde, indicating the formaldehyde removal performance of aldehyde coatings.

(a) Dissolve 0.25 g AHMT in 50 mL, 0.1 mol/L hydrochloric acid solution (a.q.) to obtain AHMT solution;
(b) Cut the qualitative filter paper into pieces of 1 cm × 1 cm and add a drop of 50 mg/kg formaldehyde solution using a pipetting gun;
(c) Get the filter paper with clean tweezers, scrape the filter paper gently to remove the excess fluid, and paste it on the aldehyde removal hybrid film. The filter paper and hybrid film are placed in a large culture dish and encapsulated in a transparent seal bag. Let stand for one hour at room temperature;
(d) Take out filter paper and drop 5 mol/L KOH solution and AHMT reagent on the filter paper. Let stand for 5 min and observe the color changes, and take photos with single lens reflex camera.

#### 2.5.2. FTIR of Hybrid Films

**Figure 2:**
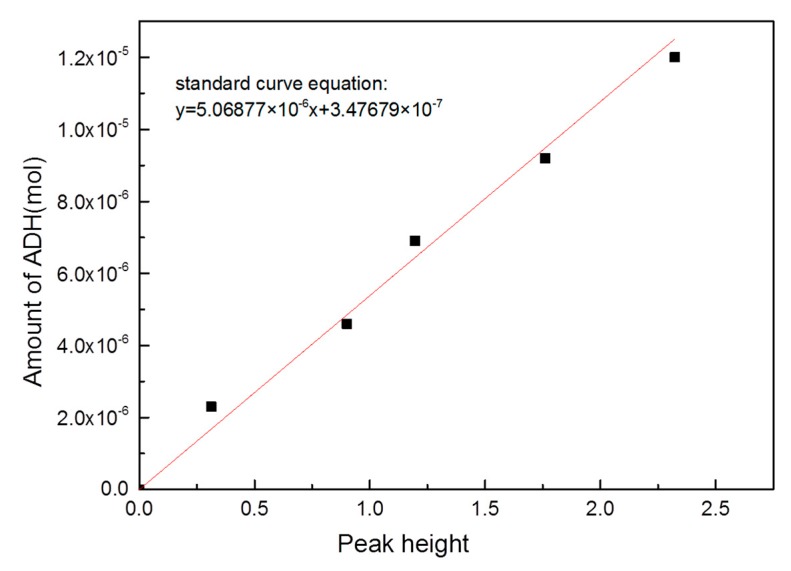
Peak values at approximately 3313 cm^−1^ were regarded as a function of the –N–H concentration in hybrid films. The plot is used to determine the –N–H concentration or the amount of adipicdihydrazide (ADH) in the hybrid film after various reaction times of formaldehyde degradation.

Infrared analysis was conducted using a Spectnlm2000 spectrometer (Perkin Elmer Co., Waltham, MA, USA) equipped with film test bracket. The Fourier transform IR spectrum of the hybrid film was recorded from 4000 to 400 cm^−1^ through 44 scans at a resolution of 4 cm^−1^. In particular, the hybrid film after 0 h, 0.5 h, and 1 h of formaldehyde degradation were measured and the peak values at 3313 cm^−1^ (N–H stretching vibration) was determined for the semi-quantitative formaldehyde degradation rate of hybrid film using the calibration curve shown in Figure 2. In order to obtain the calibration curve, a different amount of ADH was added to 0.12 g of potassium bromide, and the mixture was compressed using a tablet press. Then, the samples were analyzed using an infrared spectrometer. The intensity of the absorption peak of the amine groups and the content were employed for the linear calibration curve.

#### 2.5.3. Other Measurements

The UV–Vis spectra were obtained using a Varian Cary 500 UV–Vis spectrophotometer. SEM micrographs were obtained by Nova Nano-SEM 430 at 10 kV. Thermo gravimetric analysis (TGA) was conducted with a Netzsch TG 209 thermo-analyzer from room temperature to 600 °C with a heating rate of 10 °C/min under an N_2_ atmosphere. DSC analysis was conducted with a NETZACH DSC 204 (Netzsch, Selb, Germany), heating from −25 to 60 °C at a rate of 10 °C/min in an N_2_ atmosphere.

## 3. Results and Discussion

### 3.1. Characterization of ADH/P(MMA-BA-MAA) Hybrid Latex

**Figure 3:**
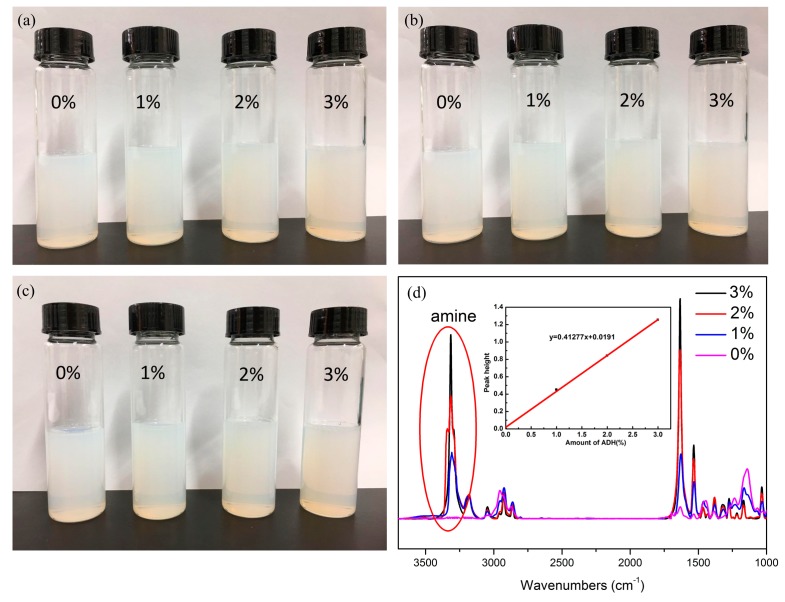
A diagram of stability of polymer emulsion with different amounts of ADH addition at different times: (**a**) 0 h, (**b**) 12 h, (**c**) 24 h. (**d**) FTIR spectrum of ADH/poly(methyl methacrylate-butyl acrylate-methacrylic acid) (P(MMA-BA-MAA)) hybrid emulsion.

Figure 3 presents the stability of the hybrid latex with different amounts of ADH (0%, 1%, 2%, 3%). It shows that the polymer latex is stable and homogeneous without any ADH sediment or polymer particles aggregation, which means ADH will not precipitate from the polymer latex or destabilize the latex by depletion force or bridging.

**Figure 4:**
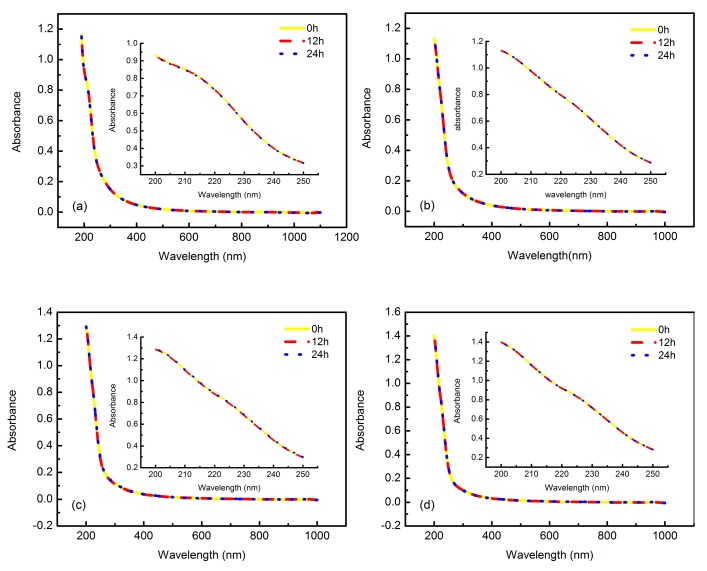
Correlation between absorbance of latex and time: (**a**) 0%, (**b**) 1%, (**c**) 2%, and (**d**) 3%.

To further evaluate the stability of the polymer latex with ADH addition, the UV–Vis absorption experiment of the polymer latex was performed. According to the Lambert Bill law, the absorbance is independent of the concentration of the emulsion particles in the dispersion. Therefore, the ratio of light absorbed is determined by the concentration of latex particles [35,36]. The absorbance curves of latex mixing with different amounts of ADH for 0, 12, and 24 h were measured. The results are shown in Figure 4. It shows that the absorbance curves with different amounts of ADH addition after 0, 12, and 24 h are completely overlapped, which further proves the excellent compatibility of ADH and polymer latex and the outstanding storage stability of the hybrid latex.

### 3.2. Characterization of ADH/P(MMA/BA/MAA) Hybrid Films

**Figure 5:**
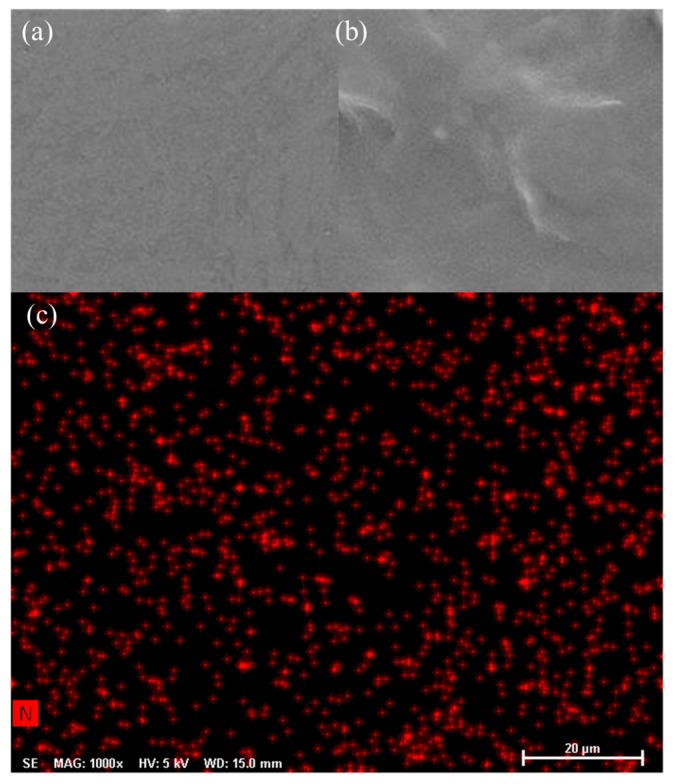
SEM micrographs of fracture surface of ADH/P (MMA-BA-MAA) hybrid films: (**a**) 0 wt% ADH, (**b**) 5 wt% ADH; (**c**) Mapping micrographs of the surface of ADH/P(MMA-BA-MAA) hybrid films, 5 wt% ADH.

SEM was adopted to investigate the effect of ADH addition on the film formation of polymer latex. Mapping was used to observe the distribution of ADH on the film. Figure 5a,b present the SEM micrographs of fracture surfaces of the neat P(MMA-BA-MAA) film and the P(MMA-BA-MAA)/ADH hybrid film. It shows that the fracture surface of the neat P(MMA-BA-MAA) film (Figure 5a) is rather smooth and that of the P(MMA-BA-MAA)/ADH hybrid film (Figure 5b) is relatively rough. Figure 5c indicates the existence of the N element in the P(MMA-BA-MAA)/ADH hybrid film, which verifies the presence of ADH compounds in the hybrid film. Moreover, it can be seen in the mapping that the ADH is dispersed homogeneously in the polymer matrix.

### 3.3. Detection of Formaldehyde Degradation

#### 3.3.1. Reaction Mechanism of Formaldehyde and ADH

**Figure 6:**
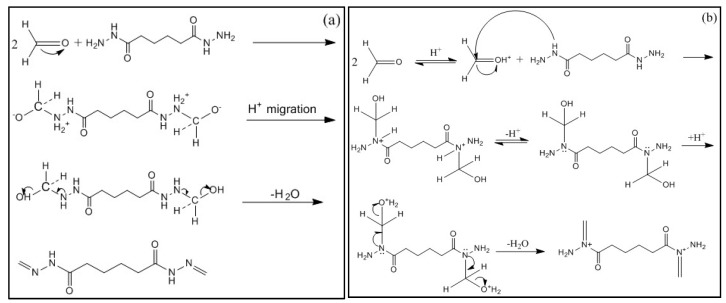
Reaction principle diagrams of formaldehyde with ADH, (**a**) primary amine and (**b**) secondary amine.

Figure 1 demonstrates the overall process of the nucleophilic reaction of formaldehyde and amine group (primary and secondary amines) to form an imino-based functional group. Formaldehyde is consumed during the reaction and free formaldehyde in the air is thus reduced. Primary amines are first targeted by alpha-hydrogen on the formaldehyde molecule and then secondary amines, as primary amines are more active than secondary amines [37,38]. One molecule of water is removed and a carbon-nitrogen double bond functional group is formed (as shown in Figure 6). It is obvious that one mol of ADH could consume four mol of formaldehyde. In addition, it can also be concluded that an acidic environment promotes forward reaction, and vice versa.

#### 3.3.2. Qualitative Test of the AHMT-RGB Method

**Figure 7:**
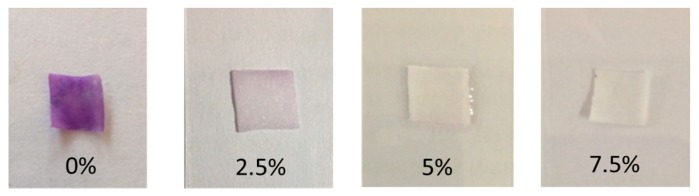
AHMT qualitative tests results for degradation of formaldehyde films.

In order to understand the effect of hybrid films on formaldehyde degradation, the residual formaldehyde was detected. AHMT was applied to qualitatively measure the residual quantity of formaldehyde because the color darkened with the increase of free formaldehyde [39]. The method has advantages including simple operation, visual phenomenon, and low requirements for experimental instrument. As shown in Figure 7, the color of the sample without any ADH is deep purple, indicating the highest concentration of free formaldehyde among the four samples. ADH is an amidic material that reacts with formaldehyde. With ADH addition, the color of the samples becomes lighter and lighter, showing that the amine group has a significant effect on formaldehyde degradation, and the efficiency increases with the increase of ADH. Taking into account its influence on hybrid films, 5% is determined as the best additive content.

#### 3.3.3. Semi-Quantitative Measurement

**Figure 8:**
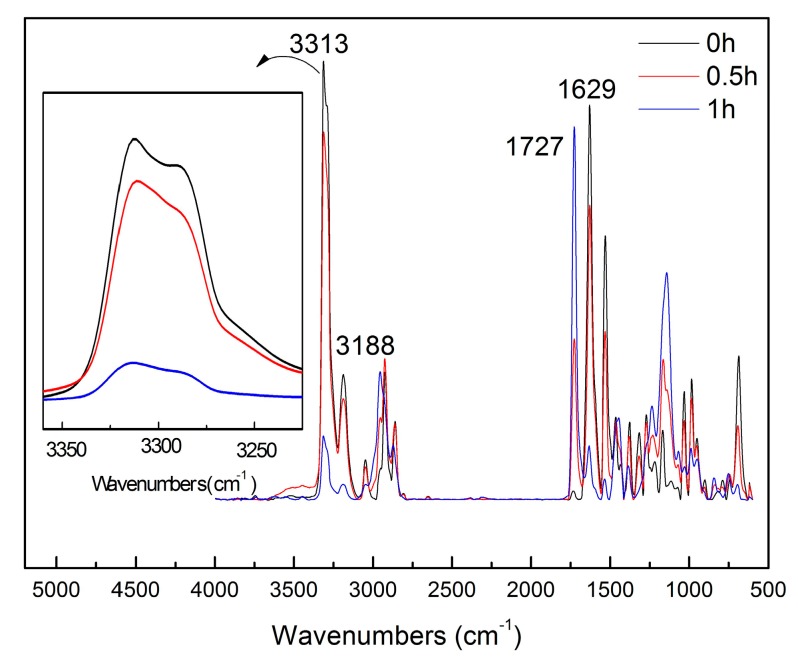
FTIR spectra of ADH/P(MMA-BA-MAA) hybrid films after different absorption time.

The advantage of the AHMT method in determining the formaldehyde removal rate of hybrid film is speed. However, the quantitative removal rate of formaldehyde could not be obtained, so a semi-quantitative measurement was carried out. According to the linear relationship of the amine group’s peak height and the concentration of aldehyde groups in Figure 2, the semi-quantitative measurement was achieved by analyzing FTIR spectra of the hybrid films after different formaldehyde adsorption time [40,41,42,43]. The results are shown in Figure 8.

As shown in Figure 8, the amino absorption peaks of ADH are at 3313 and 3188 cm^−1^ respectively, and the absorption peak at 1629 cm^−1^ corresponds to –CO–NH–. More importantly, a new peak appears at 1727 cm^−1^, which could be attributed to the characteristic absorption peak of –N=CH_2_ resulting from the reaction of amino and formaldehyde. It is obvious that with the reaction between amine and aldehyde, the intensity of amine (3313 cm^−1^) becomes weaker and weaker, but that of –N=CH_2_ increases, indicating that the reaction of amine and formaldehyde occurs. Therefore, it is reasonable that the peak at 3313 cm^−1^ is used for quantitative analysis of formaldehyde degradation. The consumption rate of the ADH sample could be calculated based on the calibration curve of peak heights and the concentration of –N–H in the hybrid film, as shown in Figure 2.

Figure 8 shows the peak heights at 3313 cm^−1^ of the ADH decrease with adsorption time, and the data are listed in Table 2. From Table 2, we can obviously see that the peak height of N-H stretching vibration is 0.63727 at the 0 h, and after degrade formaldehyde for 1 h, the peak height decreases to 0.09174. According to the calibration curve in Figure 2, the consumption rate of ADH is 2.765 × 10^−6^ mol/h in the process of formaldehyde degradation. As discussed in Section 2.5, it is known that one mol of ADH consumes four mol of formaldehyde, so the degradation rate of formaldehyde is 1.106 × 10^−5^ mol/h. Given the volume of the tested sample, that is, 1.07 × 10^−7^ m^3^, the rate of formaldehyde degradation is 1.034 × 10^2^ mol/(h·m^3^) for the hybrid film with 5 wt% of ADH.

### 3.4. Measurement of Thermodynamic Performance

**Figure 9:**
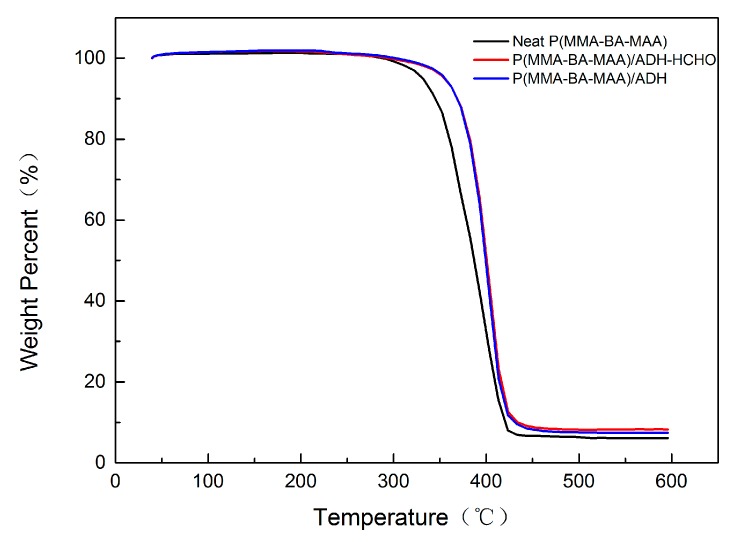
Thermo gravimetric analysis (TGA) curves for neat P(MMA-BA-MAA), P(MMA-BA-MAA)/ADH, and P(MMA-BA-MAA)/ADH-formaldehyde (HCHO) hybrid films.

TGA measurements were performed to study the thermodynamic stability of the hybrid films. Figure 9 shows the TGA curves of neat P(MMA-BA-MAA), P(MMA-BA-MAA)/ADH, and P(MMA-BA-MAA)/ADH-HCHO hybrid films, and the corresponding data are listed in Table 3. As shown in Figure 9 and Table 3, the half-degradation temperature (*T*_0.5_) of neat P(MMA-BA-MAA) is 395.8 °C, while *T*_0.5_ of the hybrid film with 5 wt% of ADH increases to 403.2 °C. The thermodynamic stability of the hybrid film is further improved after it reacted with formaldehyde, and the *T*_0.5_ increases to 404.1 °C. The residual carbon of the hybrid films after pyrolysis in an N_2_ atmosphere also increases with the addition of ADH and reaction with formaldehyde. The results suggest that ADH additive effectively improves the thermal stability of P(MMA-BA-MAA) materials, and the P(MMA-BA-MAA)/ADH hybrid film with fixed formaldehyde has better thermal stability. This might be explained by the dehydration condensation reaction of aldehyde groups and amine groups, where formaldehyde is captured in the polymer by chemical reactions, resulting in the increase of decomposition temperature.

**Figure 10:**
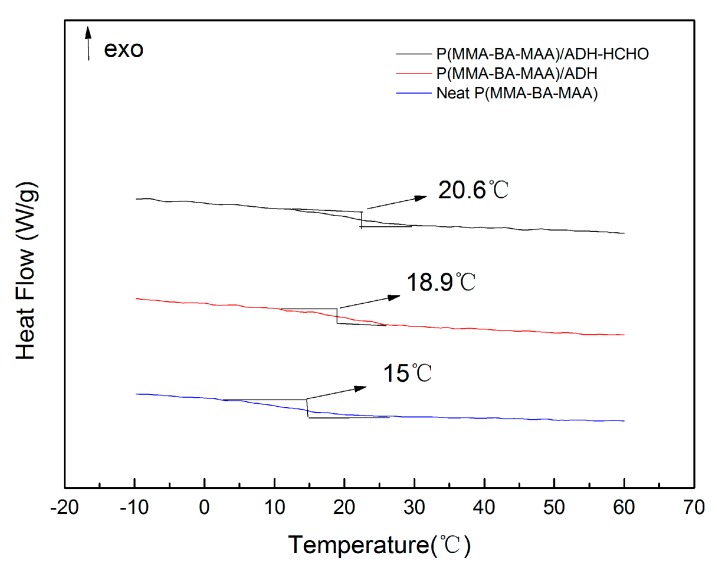
DSC curves of neat P(MMA-BA-MAA), P(MMA-BA-MAA)/ADH, and P(MMA-BA-MAA)/ADH-HCHO hybrid films.

DSC measurements were taken in the glass transition region for all the samples. The DSC curves are displayed in Figure 10 and the DSC data of hybrid films are listed in Table 3. It shows that the glass transition temperature (*T*_g_) of neat P(MMA-BA-MAA) is 15 °C, while *T*_g_ of the hybrid films are 18.9 and 20.6 °C for P(MMA-BA-MAA)/ADH and P(MMA-BA-MAA)/ADH-HCHO hybrid films, respectively, which is 3.9 and 5.6 °C higher, respectively, than that of neat P(MMA-BA-MAA) films. It might be explained by the effect of the nanoparticles of ADH and the interaction of the reaction products of ADH and formaldehyde, which lowers the thermal movement of the polymer chains [44].

## 4. Conclusions

A simple and versatile route for industrial scale synthesis of homogeneous ADH/polymer hybrids with excellent performance of formaldehyde degradation is proposed, using the semi- continuous emulsion polymerization process and ultrasonic cell grinder. ADH compounds could be dispersed evenly in the polymer matrix because of the two amine groups and its excellent hydrophilicity. Compared with neat polymers, the thermal properties of hybrid films are significantly improved. Besides, it demonstrates excellent ability to degrade formaldehyde-the efficiency of degradation is up to about 100% and the rate of formaldehyde degradation is 1.034 × 10^2^ mol/(h·m^3^) for the hybrid film with 5 wt% ADH. The hybrid latexes will be used as the binder of functional waterborne coatings for unbound formaldehyde adsorption and degradation in habitable spaces.

## Figures and Tables

**Table 1:** Recipe for emulsion polymerization. MMA—methyl methacrylate; BA—butyl acrylate; MAA—methacrylic acid; HD—hexadecane; SDS—sodium dodecyl sulfate; KPS—potassium persulfate.

Ingredient		Weight (g)
monomer	MMA	50
	BA	48
	MAA	2
co-stabilizer	HD	2
emulsifier	SDS	2
Initiator	KPS	0.5
Deionized water	DI water	150

**Table 2:** Absorption peak heights of -NH-NH_2_ in adipicdihydrazide (ADH) after different absorption times.

	Time	S_1(0 h)_	S_2(0.5 h)_	S_3(1 h)_
Parameter				
Peak heights		0.63727	0.53486	0.09174

**Table 3:** Thermal properties of poly (methyl methacrylate-butyl acrylate-methacrylic acid) (P(MMA-BA-MAA)), P(MMA-BA-MAA)/ADH, and P(MMA-BA-MAA)/ADH-HCHO TGA-thermo gravimetric analysis.

Sample	TGA Data		DSC Data (°C)		
	*T*_0.5_ (°C)	Residual Carbon (%)	*T*_onset_	*T*_end_	*T*_g_
Neat polymer	395.8	4.8	6.2	18.4	15
ADH/polymer hybrid	403.2	6.5	14	25.1	18.9
Hybrid film after adsorbing HCHO	404.1	6.8	15.4	26.8	20.6
